# Building an Easy-to-Assemble, Low-Cost Phonomicrosurgery Dissection Model: A Technical Report From the University of Cartagena

**DOI:** 10.7759/cureus.76194

**Published:** 2024-12-22

**Authors:** Steven Osorio, Catalina Pachon, Alejandro Uribe Escobar

**Affiliations:** 1 Otolaryngology and Laryngology, Hospital Serena Del Mar - Universidad De Cartagena, Cartagena, COL; 2 Otolaryngology, Universidad De Cartagena, Cartagena, COL

**Keywords:** laryngeal microsurgery, laser microsurgery for benign and malignant laryngeal tumors and airway stenosis, simulation medicine, simulation trainer, teaching by simulation

## Abstract

In otolaryngology, training often involves simulation in animal specimens, human cadavers, and artificial models to facilitate learning surgical procedures, reducing the time needed to acquire essential skills. Simulated training has become integral to medical education, particularly in microsurgical techniques, such as microlaryngeal surgery. These procedures, also known as phonomicrosurgery, are performed on the vocal folds using microscopic visualization and precision instruments with long shafts and millimetric tips. In otolaryngology programs, ear surgeries were the first to be incorporated into temporal bone laboratories, making such training a standard requirement for all teaching institutions. Similarly, laryngeal models have been developed to support phonomicrosurgery training. However, these models are often expensive or technically complex to assemble, limiting their accessibility for many institutions. This report provides a step-by-step guide to building a phonomicrosurgery model designed for practicing various surgical techniques using porcine or human cadaveric larynges. The model reduces costs to approximately 20 USD, is constructed from readily available materials, and can be easily assembled by untrained personnel. It offers an accessible solution for enhancing phonomicrosurgical skills in otolaryngology residency programs.

## Introduction

In recent decades, increased emphasis on patient safety in resident training programs has reduced opportunities for hands-on learning in the operating room. Nevertheless, the technical skills required for laryngeal microsurgery remain critical for achieving optimal patient outcomes and are often challenging to teach in vivo. To address this gap, simulation-based training has become a cornerstone of medical education, offering residents the opportunity to develop and refine surgical techniques in a controlled, non-clinical environment [1-3].

Phonomicrosurgery aims to restore or enhance vocal quality by excising lesions such as nodules, polyps, cysts, premalignant lesions, or other abnormalities while preserving the surrounding tissue and preventing scarring of the vocal folds. The traditional training methods of these procedures have developed various models, including ovine, porcine, bovine, canine, human cadaveric, and synthetic larynges. Among these, the human cadaveric larynx, while ideal, is often difficult to obtain due to regulatory and logistical constraints, which vary by country. In contrast, porcine larynges are widely available, anatomically comparable, and present minimal limitations, making them a practical alternative for simulation training [2,4-6].

Training in an ex vivo porcine laryngeal model has been shown to require approximately 10 sessions for an inexperienced otolaryngology resident to improve their skills in microlaryngeal surgery [6]. Building on the utility of such models, various laryngeal dissection stations have been described in the literature for training purposes; however, none are commercially available. Many of these stations are characterized by high costs, excessive complexity, or reliance on specialized materials such as polycarbonate, wood, or steel, which may not be easily accessible [2,7-9]. To address these limitations, we developed an easy-to-assemble, low-cost, and replicable laryngeal dissection station specifically designed for practicing phonomicrosurgical techniques in porcine larynges. This report provides a step-by-step guide for constructing the station using readily available and long-lasting materials, primarily polyvinyl chloride (PVC) pipes, screws, and other items commonly found in local hardware stores.

## Technical report

**Figure 1:**
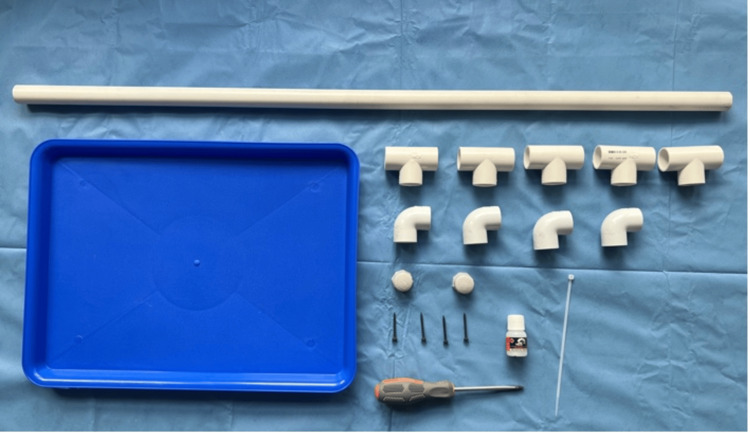
Materials required for assembling the laryngeal suspension model

The laryngeal suspension frame was constructed using 26 mm (¾ inch) PVC pipes and the following components: a 35 x 45 cm plastic tray, five PVC equal Tee connectors (T-connector), four PVC 90° elbow connectors, one meter of PVC ¾-inch diameter pipe, two PVC ¾-inch pipe caps, four 1 ½-inch screws, a screwdriver, PVC solvent cement (adhesive), and adjustable plastic clamps (Figure 1). The list of the item's costs can be found in Table 1.

**Table 1:** List of items and prices PVC, polyvinyl chloride

Item	Price (USD)
1 Plastic tray 35 x 45 cm	3,99
5 PVC equal Tee ¾ inch	1,70
4 PVC 90° elbow connectors ¾ inch	1,04
PVC ¾-inch diameter pipe - 1 m	2,50
2 PVC ¾-inch pipe caps	1,06
4 1 ½-inch screws	0,6
PVC solvent cement	3,15
Adjustable plastic clamps	6,04
Total	20,08

**Figure 2:**
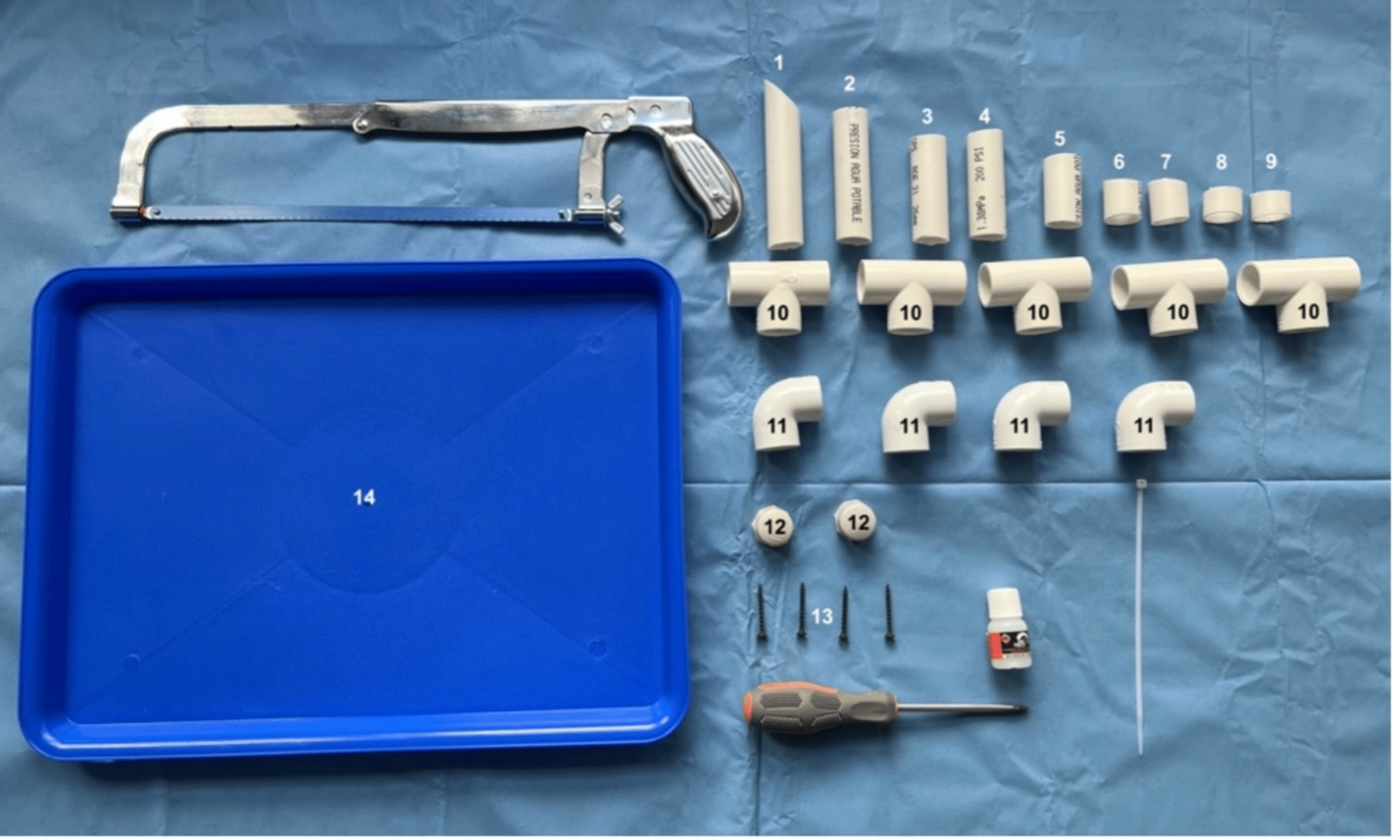
PVC pipe segments of varying lengths customized for the suspension model PVC, polyvinyl chloride

**Figure 3:**
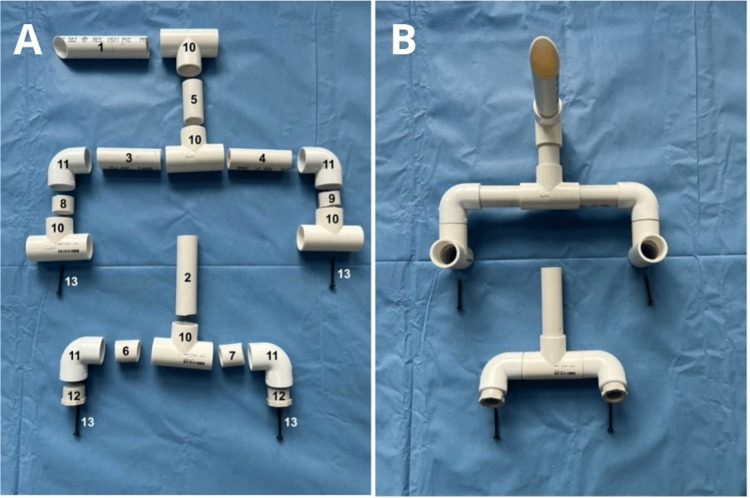
Arrangement of PVC pipe segments and connectors before assembly PVC, polyvinyl chloride

The pipe was cut into nine segments using a handsaw: one piece measuring 13 cm in length with a 45° beveled angle at one end to simulate the structure of a rigid laryngoscope (#1), one piece measuring 10 cm (#2), two pieces measuring 8 cm each (#3 and #4), one piece measuring 5 cm (#5), two pieces measuring 4 cm each (#6 and #7), and two smaller pieces measuring 3 cm each (#8 and #9) (see Figure 2 and Figure 3).

**Figure 4:**
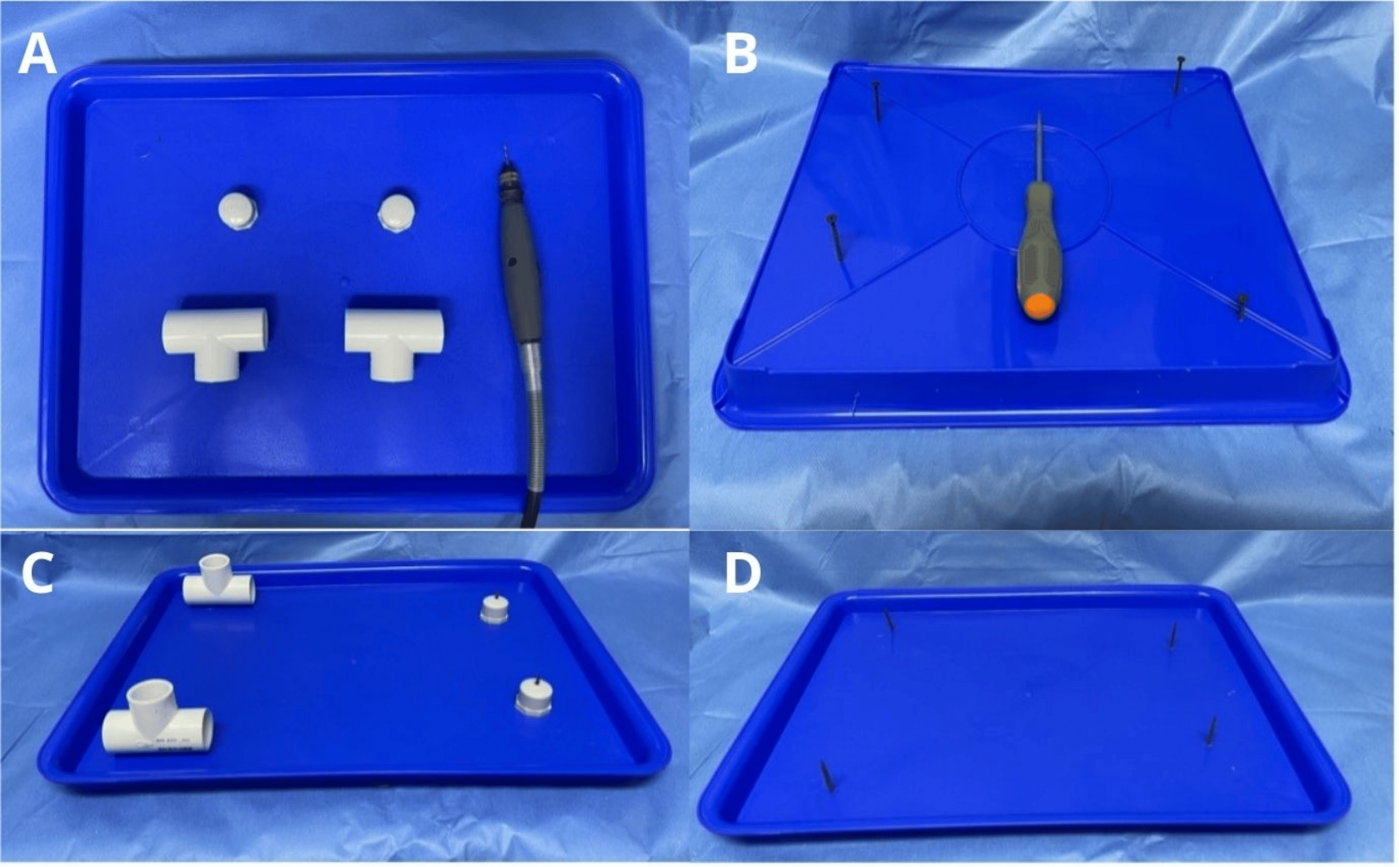
Step-by-step process for drilling holes to secure and stabilize the model

Before assembling, holes were drilled in the center of two equal T-connectors and the two pipe caps to accommodate screws. Corresponding holes were also drilled into the plastic tray at distances of 15 cm and 25 cm, respectively (see Figure 4).

**Figure 5:**
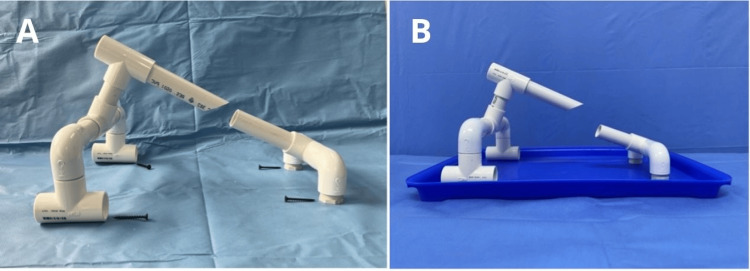
Fully assembled PVC model secured to the plastic tray PVC, polyvinyl chloride

After drilling, the assembly process began. The numbered PVC segments were secured using PVC solvent cement, forming the framework. Once assembled, the framework was attached to the plastic tray using screws for stabilization (see Figure 5).

**Figure 6:**
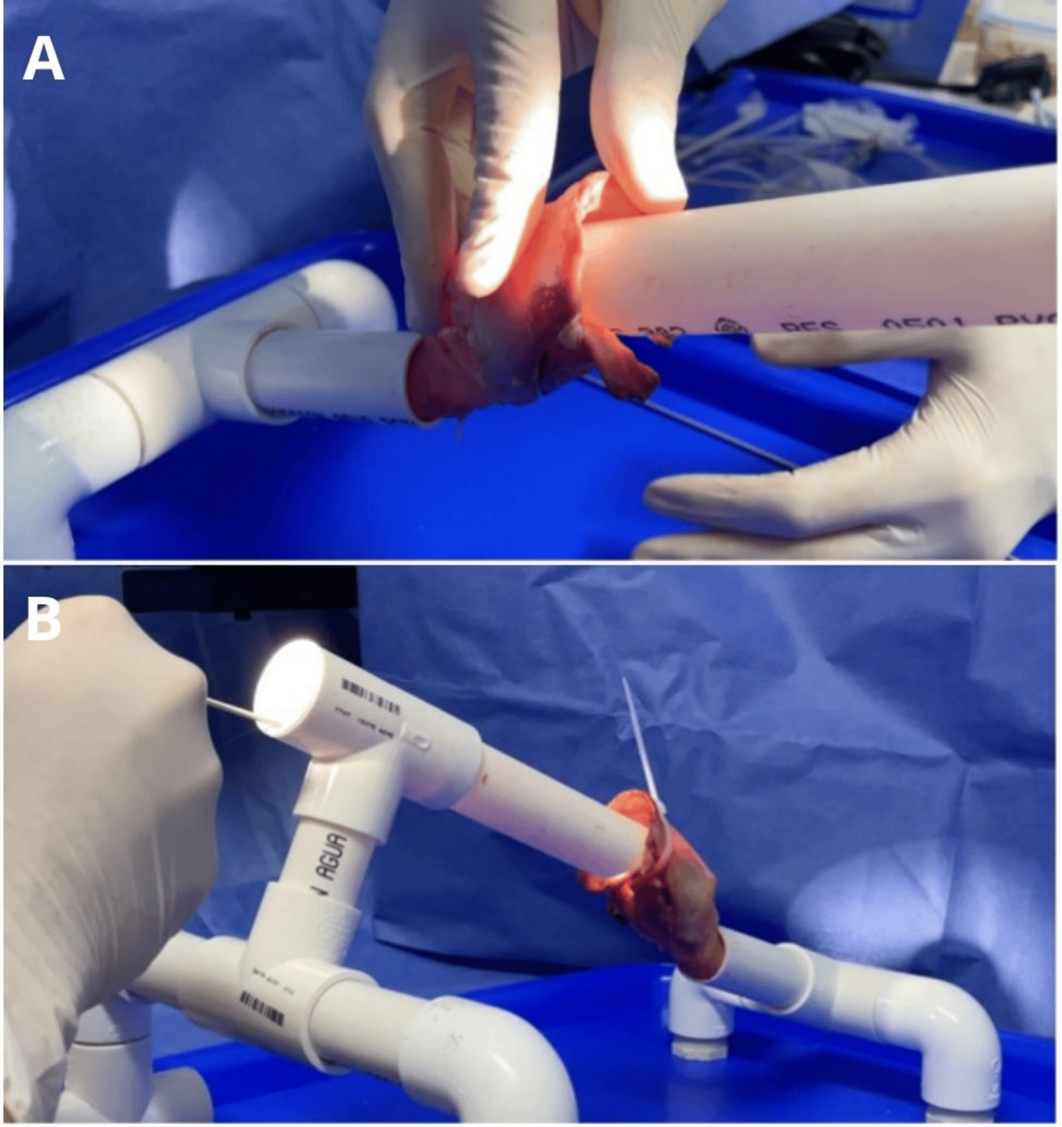
Mounting the laryngeal specimen onto the model for phonomicrosurgery practice

The ex vivo porcine laryngeal specimen was mounted onto the suspension model and secured to the PVC tube using adjustable plastic clamps (see Figure 6). Sometimes it is necessary to remove some of the redundant arytenoid cartilage to improve the vocal fold exposition.

**Figure 7:**
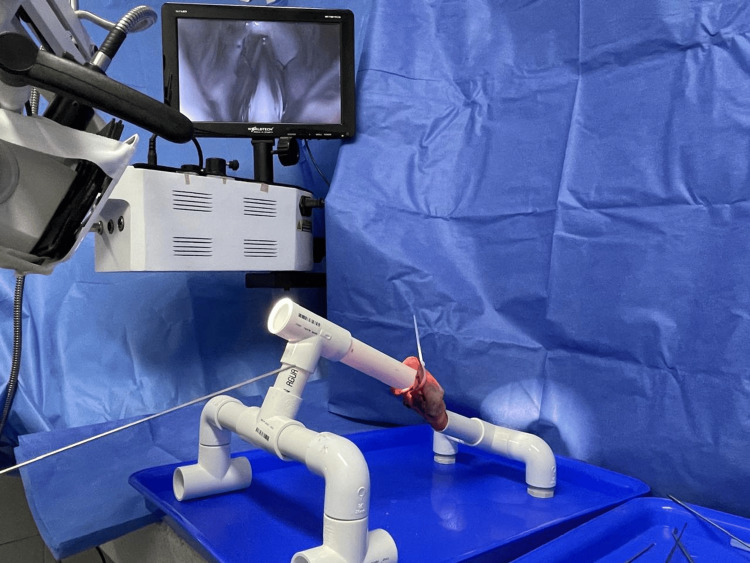
Demonstration of the model setup in the university laboratory for phonomicrosurgery training

This model was adapted for use in the temporal bone laboratory at the University of Cartagena. It was paired with a microscope equipped with a 400 mm focal length, a camera, and a microlaryngeal instrument set (see Figure 7 and Video 1). In the context of an annual laryngeal course, we conducted a pilot study with eight otolaryngology residents to assess the ease of setting up the specimen and the simulator. The average time to mount the porcine specimen onto the simulator was 35 seconds (24-144 seconds) while adjusting the microscope for proper laryngeal exposure took an average of 128 seconds (90-170 seconds).

**Video 1:** Step-by-step guide for constructing the station

## Discussion

Simulation-based training has become a cornerstone of modern medical education, forming a critical component of otolaryngology residency curricula. In the field of laryngology, practical experience with intricate procedures such as phonomicrosurgery is imperative for residents to develop the necessary skills. Microlaryngeal surgery, which includes the excision of polyps, cysts, nodules, premalignant lesions, and other epithelial or subepithelial vocal fold diseases, demands exceptional dexterity. Surgeons must operate with both hands simultaneously at a focal distance of 350 to 415 mm while addressing lesions that measure only a few millimeters [10]. According to Ghirelli et al, otolaryngology residents require approximately 10 sessions with an ex vivo porcine laryngeal model to achieve significant progress in microlaryngeal surgical skills [6]. With the need to improve the outcomes in in vivo patients, an otolaryngology residency program must have a microlaryngeal dissection station.

The materials and costs of laryngeal phonomicrosurgery simulation sets described in the literature have shown considerable variability. In 2004, Mohamed et al. introduced a model designed with steel, at a cost of approximately $1,000 USD [8]. Similarly, Verma et al. presented a laryngeal surgery simulator made of stainless steel, with an estimated cost of $800 USD [2]. In the same context, Mattioli et al. described and validated a polycarbonate-based laryngeal dissection station for practicing laser and cold knife microlaryngeal surgery using an ex vivo porcine model [9]. This station was patented and has an approximate cost of $300 Euros, its assembly and the required materials are more complex compared to other available models.

Alternative designs have been developed to offer more affordable options. Holliday et al. published a model constructed from PVC, incorporating a wooden base and copper pipes, with a total cost of under $24 USD, and could also be adapted for use with a standard stainless-steel laryngoscope [4]. In 2015, Zambricki et al. described a compact PVC-based practice station that utilized grape specimens embedded in a gelatin mold, with a cost of just $20 USD [11]. Our model shares some features with this station and facilitates the use of high-fidelity models, such as porcine ex vivo larynx specimens. The comparisons of the laryngeal simulator reported can be found in Table 2.

**Table 2:** Comparison of laryngeal simulators reported in the literature

Author	Material	Cost	Specimens
Mohamed et al. (2004)	Steel	$1,000 USD	Human cadaveric larynx
Verma et al. (2010)	Stainless steel	$800 USD	Canine Larynx
Holliday et al. (2015)	PVC, wooden base, copper pipes	$24 USD	Grape
Zambricki et al. (2016)	PVC, laryngoscope	$20 USD	Grape
Mattioli et al. (2017)	Polycarbonate-based, laryngoscope	$300 Euros	ex vivo lamb larynx
Maguire et al. (2018)	3D-printed	$78 USD	ex vivo porcine larynx
Yu et al. (2020)	Wood, laryngoscope	No data	ex vivo porcine larynx
University of Cartagena Laryngeal Lab	PVC	$20 USD	ex vivo porcine larynx

In recent years, with the advent of 3D printing, Maguire et al. developed a 3D-printed laryngoscope adaptable to porcine larynxes. This model costs approximately $78 USD, though it requires access to a 3D printer, which is not universally available [12]. In contrast, Yu et al. (2020) designed a model primarily made of wood, which is inexpensive and suitable for use with porcine specimens [7]. However, this design requires the assistance of an individual with woodworking skills to construct the station.

In this context, our model offers a valuable addition to the existing range of simulators. With a total cost of approximately $20 USD, it is constructed from commonly available materials, is easy to clean, and ensures accessibility for institutions with limited budgets. This station can be assembled in approximately two minutes and is designed for use with basic microlaryngeal surgical tools and a 350 to 400 mm microscope lens. It provides a versatile and effective training platform for residents, reinforcing the role of simulation as a fundamental component of surgical education in laryngology. This report marks the first step in validating this simulator as an assessment tool, allowing its comparison with existing microlaryngeal simulation stations.

## Conclusions

The phonomicrosurgery station described is useful for practicing microlaryngeal surgery in ex vivo porcine larynx, thereby improving phonosurgical skills. To optimize patient outcomes in voice surgery within residency training institutions, it is essential to address the learning curve through systematic practice using a microlaryngeal simulator. Just as temporal bone laboratories are a standard component of otolaryngology training programs, it has now become essential to have phonomicrosurgery dissection stations in training centers. Compared to the stations described in the literature, ours features an easy setup and a low-cost design, utilizing materials readily available at a local hardware store. This makes it a potentially suitable option for otolaryngology programs in low-income countries, especially when compared to more complex stations described in the literature. Future work should focus on obtaining valid evidence for this model.
